# Geographical distribution and pathogenesis of ticks and tick-borne viral diseases

**DOI:** 10.3389/fmicb.2023.1185829

**Published:** 2023-05-24

**Authors:** Taif Shah, Qian Li, Binghui Wang, Zulqarnain Baloch, Xueshan Xia

**Affiliations:** ^1^Faculty of Life Science and Technology, Kunming University of Science and Technology, Kunming, Yunnan, China; ^2^Provincial Center for Molecular Medicine, Kunming, China

**Keywords:** ticks, tick-borne viruses, epidemiology, pathogenesis, manifestations

## Abstract

Ticks are obligatory hematophagous arthropods that harbor and transmit infectious pathogens to humans and animals. Tick species belonging to *Amblyomma*, *Ixodes*, *Dermacentor*, and *Hyalomma* genera may transmit certain viruses such as Bourbon virus (BRBV), Dhori virus (DHOV), Powassan virus (POWV), Omsk hemorrhagic fever virus (OHFV), Colorado tick fever virus (CTFV), Crimean-Congo hemorrhagic fever virus (CCHFV), Heartland virus (HRTV), Kyasanur forest disease virus (KFDV), etc. that affect humans and certain wildlife. The tick vectors may become infected through feeding on viraemic hosts before transmitting the pathogen to humans and animals. Therefore, it is vital to understand the eco-epidemiology of tick-borne viruses and their pathogenesis to optimize preventive measures. Thus this review summarizes knowledge on some medically important ticks and tick-borne viruses, including BRBV, POWV, OHFV, CTFV, CCHFV, HRTV, and KFDV. Further, we discuss these viruses’ epidemiology, pathogenesis, and disease manifestations during infection.

## 1. Overview

Ticks are obligatory hematophagous parasites, harboring and transmitting infectious pathogens to humans and animals in temperate North America, Europe, and Asia. Ticks are responsible for more than 95% of vector-borne diseases in the United States (Rosenberg et al., 2018). Tick-borne diseases have a significant economic impact, especially in developing countries (Estrada-Peña and Salman, 2013). Tick-borne diseases affect approximately 80% of the world’s cattle population, with an estimated cost of around $13.9–18.7 billion (De Castro et al., 1997). For country like Tanzania, the economic losses due to tick-borne disease has been quantified to be $364 million, with an estimated mortality of 1.3 million cattle (Kivaria, 2006). In the United States, the Powassan virus (POWV) transmission has been seen as evidence of an emerging trend, spreading in new areas at a comparable rate (Hermance and Thangamani, 2017). Tick-borne diseases, particularly Crimean-Congo hemorrhagic fever (CCHF), are persistent problems at the One Health interface between humans, wildlife, and the environment (Anderson and Armstrong, 2012; Sorvillo et al., 2020). These conditions are often ignored, and receive less attention and dedicated public health funding than directly transmitted zoonotic viruses such as the recent COVID-19 pandemic (WHO, 2022b). Tick vector control’s ability to reduce tick populations is limited by the availability of large-scale mitigation strategies and preventive control measures, demanding additional research to develop effective tick control strategies for human and wildlife (Eisen and Stafford, 2021). Current prevention and clinical treatment are limited, despite their severe prognosis and high fatality rate. Many tick-borne infections in humans are also difficult to detect and diagnose, due to the broad clinical presentation of many tick-borne diseases, the lack of reliable diagnostic tests, and the multi-tiered approaches required to confirm pathogens (Fatmi et al., 2017; Bush and Vazquez-Pertejo, 2018). In brief, the expansion of tick populations and increasing incidence of tick-borne diseases are bringing ticks to the attention of public health professionals.

**Figure 1:**
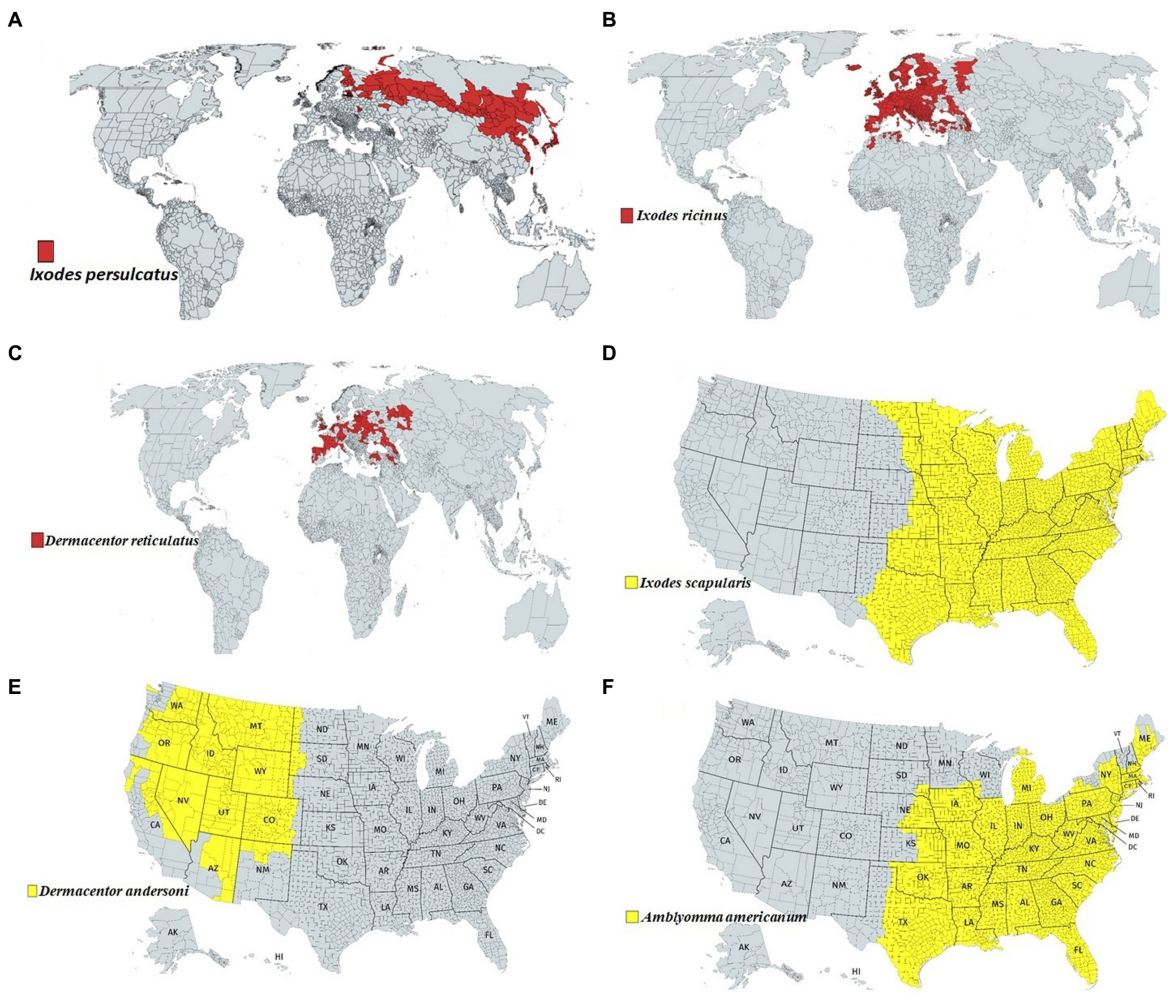
Approximate global distribution of some medically important ticks. **(A)** Ixodes persulcatus distribution in north-eastern Europe, some parts of Russia, China, Korea etc. **(B)** Ixodes ricinus in some parts of Europe and northern Africa. **(C)** Dermacentor reticulatus in some parts of Europe. **(D)** Ixodes scapularis, **(E)** Dermacentor andersoni and **(F)** Amblyomma americanum in Northern America.

**Figure 2:**
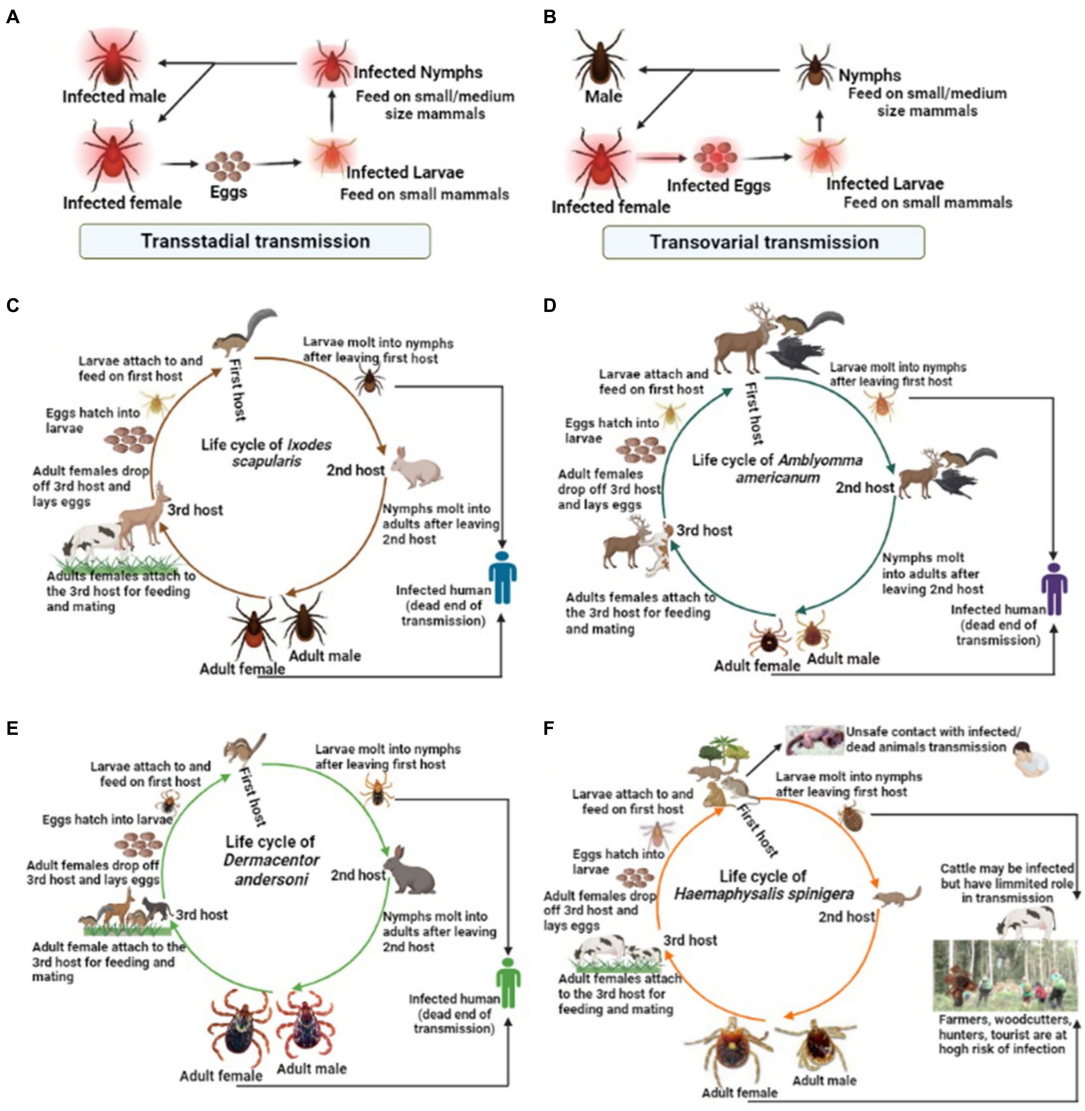
The life cycle of some medically important tick vectors and transmission routes of viruses. The two possible transmission types, i.e., **(A)** transstadial transmission and **(B)** transovarial transmission, which is critical for the virus transmission *via* infected ticks. **(C)** The life cycle of *Ixodes scapularis*, **(D)** *Amblyomma americanum*, **(E)** *Dermacentor andersoni*, **(F)** *Haemaphysalis spinigera*.

Among the tick families, i.e., hard ticks (Ixodidae), soft ticks (Argasidae), and Nuttalliellidae, Ixodidae is the most prominent family, including genera like *Amblyomma*, *Haemaphysalis*, *Ixodes*, *Rhipicephalus*, etc., and over 700 species worldwide (Brites-Neto et al., 2015; Dantas-Torres and Otranto, 2022). *Ixodes persulcatus* (Figure 1A) distribution has been reported in north-eastern Europe, some parts of Russia, China, Korea etc. (Topp et al., 2022); *I. ricinus* (Figure 1B) in Europe and northern Africa; *Dermacentor reticulatus* (Figure 1C) in some parts of Europe; *I. scapularis* (Figure 1D); *D. andersoni* (Figure 1E); and *Amblyomma americanum* (Figure 1F) in Northern America. Most members of the Ixodidae, particularly *I. scapularis* (Figure 2C), *A. americanum* (Figure 2D), *D. andersoni* (Figure 2E), and *Haemaphysalis spinigera* (Figure 2F) go through four different life stages, i.e., eggs, larvae, nymphs, and adult ticks (WHO, 2020). Among the two possible vertical transmission routes of tick-borne viruses (TBVs), transstadial transmission is the sequential passage that is critical for tick survival and life cycle completion, whereas transovarial transmission occurs when viruses are transmitted from an infected tick to their offspring, i.e., the virus remains in the egg after being laid by an infected female tick and eventually infects the offspring (Figures 2A,B; Barker and Reisen, 2019).

**Figure 3:**
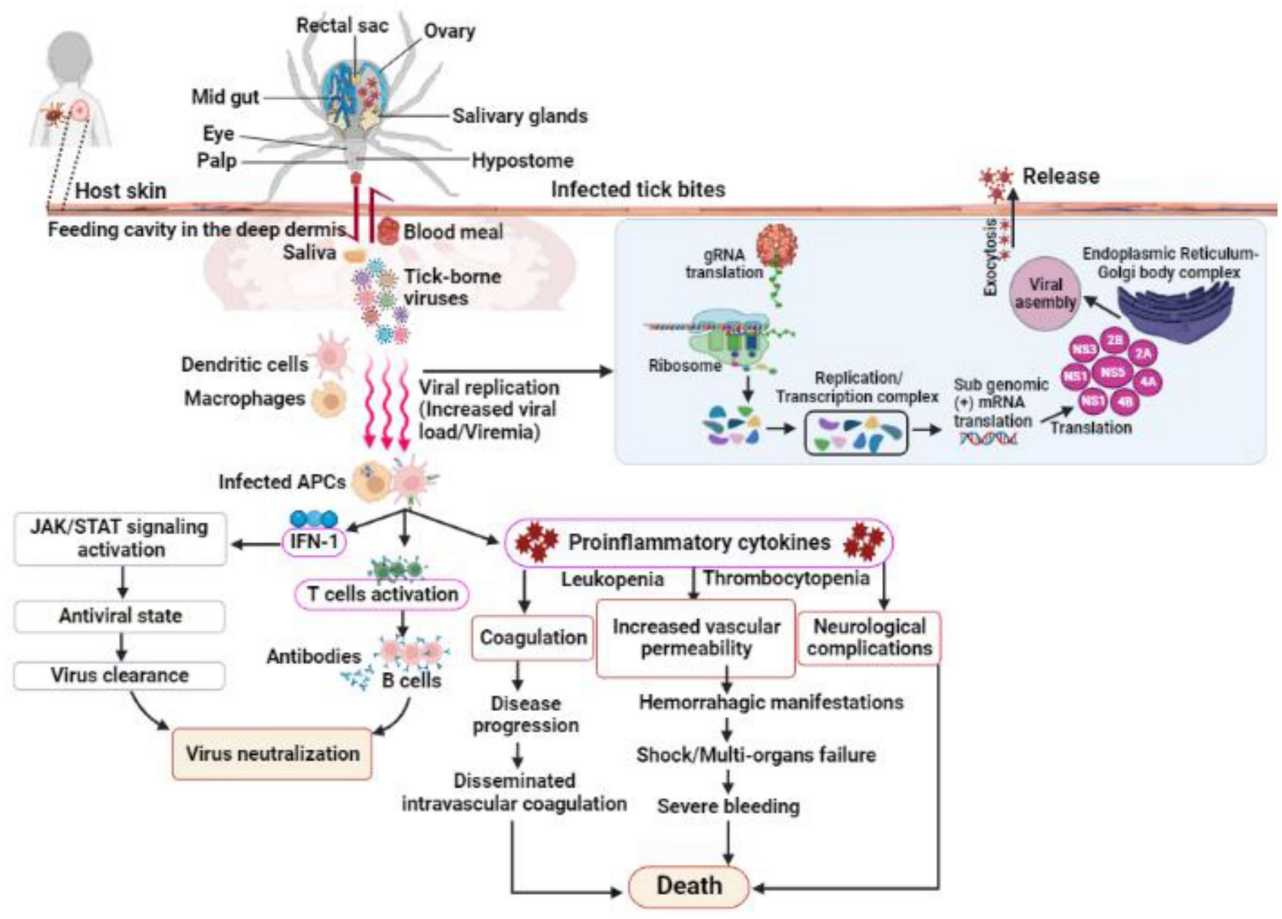
Shows the infection, replication and pathogenesis of TBVs in hosts taking *flavivirus* as an example. Ticks transmit the virus to susceptible host while feeding. TBVs enter the host cell *via* endocytosis or by fusing E protein to the heparan-sulfate and glycosaminoglycan receptors (found on vertebrates and tick cells). After entering the host cell, the virus fuses with the endosomal membrane and releases its capsid into the cytoplasm. In the infected cell cytoplasm, the viral RNA is unveiled and transcribed into proteases, which cleave the polypeptides into several NSPs, such as NS1, NS3, 2A, 2B, 4A, 4B, etc. RNA-dependent RNA polymerase and helicases work together to produce gRNA and RNAs, which are both required for viral replication and transcription. The newly synthesized viral RNA is translated, producing envelopes, capsids, membrane proteins, and other components that are then transported to the E.R-Golgi body complex for virion assembly. These virions coat the lipid bilayer completely, mature, and are exocytosed from the cytoplasm of the infected cell. Following transmission from an infected tick, the TBVs replicate and induces inflammatory responses in infected hosts, resulting in clinical signs and symptoms. These viruses use a variety of mechanisms to evade host immune responses and colonize host tissues. In some cases, the host immune responses to TBVs, particularly pro-inflammatory cytokines, can cause neurological and cardiovascular disorders during persistent infections.

TBVs use a variety of mechanisms to evade host immune responses for successful tissue colonization in humans and animals (Charrel et al., 2004; Belikov et al., 2014; Brites-Neto et al., 2015). Despite low viremia in infected vertebrates, most organ damage is caused by host inflammatory responses; for example, induced inflammatory responses in TBV infections can increase vascular permeability, neurological complications, and even death in severe cases (Bente et al., 2013; Hermance et al., 2016; Bhatia et al., 2020; Campbell and Krause, 2020; Figure 3). Nonetheless, case reports and preliminary field studies published in the last decade indicate that TBVs present in different regions are underdiagnosed or misdiagnosed due to their complex behaviors. Although the epidemiological characteristics of ticks and TBVs are well documented, understanding pathogenesis, notably host immune responses to the virus and their impact on disease outcomes, is limited. The main focus of this review is to summarize the most recent advances in TBV diseases, with a focus on emerging diseases that pose a public health threat around the world.

**Figure 4:**
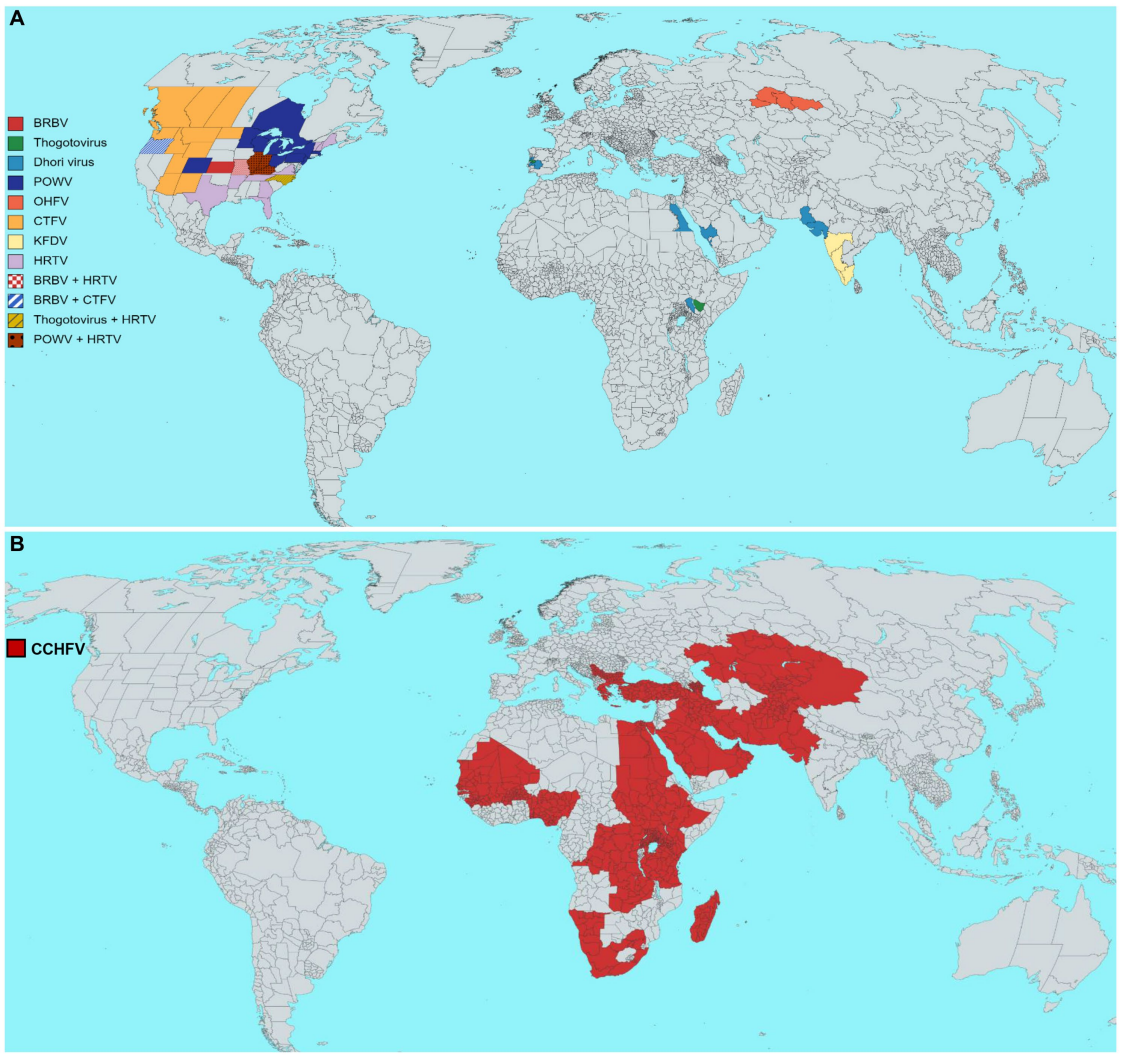
Estimated geographical distribution of selected tick-borne viruses (TBVs) of public health relevance. **(A)** Distribution of Bourbon virus (BRBV), *Dhori virus* (family *Orthomyxoviridae*, genus *Thogotovirus*), Powassan virus (POWV), Omsk hemorrhagic fever virus (OHFV), Colorado tick fever virus (CTFV), Kyasanur forest disease virus (KFDV), Heartland virus (HRTV). **(B)** The global distribution of the Crimean-Congo hemorrhagic fever virus (CCHFV). Each virus distribution has been shown according to the guidelines of WHO, the United States Centers for Disease Control and Prevention, and the European Center for Disease Prevention and Control.

This review thus emphasizes the epidemiological characteristics of some medically important tick vectors and TBVs, including Bourbon virus (BRBV), POWV, Omsk hemorrhagic fever virus (OHFV), Colorado tick fever virus (CTFV), Crimean-Congo hemorrhagic fever virus (CCHFV), Heartland virus (HRTV), and Kyasanur forest disease virus (KFDV) (Figure 4A). Details on TBVs’ classification and distribution are shown in Table 1. For this review, we have drawn data from existing literature, our basic research, and public health experience with TBV diseases. Further, we discuss the epidemiology, pathogenesis, and disease manifestations of some medically important TBVs.

**Table 1:** Characteristics of some medically important tick-borne viruses.

Virus name	Classification (family, genus)	Genome description	Distribution	Principal tick vector
Bourbon virus (BRBV)	*Orthomyxoviridae*, *Thogotovirus*	Segmented negative-stranded RNA	Eastern Asia, China, Japan	*Haemaphysalis longicornis*
Powassan virus (POWV)	*Flaviviridae*, *Flavivirus*	Positive stranded RNA	North America, Russian Federation	*Ixodes scapularis, Ixodes cookei*
Deer tick virus (DTV)	*Flaviviridae*, *Flavivirus*	Positive stranded RNA	North America	*Ixodes scapularis*
Omsk hemorrhagic fever virus (OHFV)	*Flaviviridae*, *Flavivirus*	Non-segmented positive ssRNA	Western Siberian regions	*D. reticulatus*, *Ixodes persulcatus*
Colorado tick fever virus (CTFV)	*Spinareoviridae*, *Coltivirus*	segmented double-stranded RNA	Western America, Canada	*Dermacentor andersoni*
Crimean Congo hemorrhagic fever virus (CCHFV)	*Bunyaviridae*, *Nairovirus*	Tri-segmented negative-stranded RNA	Asia, Africa, Eastern Europe	*Hyalomma marginatum*
Heartland virus (HRTV)	*Bunyaviridae*, *Phlebovirus*	Tri-segmented negative-stranded RNA	North America	*Amblyomma americanum*
Kyasanur forest disease virus (KFDV)	*Flaviviridae*, *Flavivirus*	Positive stranded RNA	Southern India	*Haemaphysalis spinigera*

## 2. Bourbon virus

BRBV is a member of the *Orthomyxoviridae* family, genus *Thogotovirus*, infecting humans and animals *via* tick bites in most parts of Africa, the Middle East, and Europe (Bai et al., 2019; Fuchs et al., 2019). Viruses in this family have filamentous, rounded, enveloped, segmented negative-sense ssRNA genomes comprising six genes, encoding viral polymerase basic protein 1 (PB-1), polymerase basic protein 2 (PB-2), polymerase acidic protein (PA), a matrix protein, a nucleoprotein, and a surface glycoprotein (Kosoy et al., 2015). The increased prevalence of BRBV in the last decade emphasizes the importance of tick surveillance; for example, *Haemaphysalis longicornis* is an important tick species in eastern Asia, transmitting viral pathogens to humans and animals (Rainey et al., 2018). In Ehime, Japan, researchers isolated a novel Oz virus (a close relative of BRBV) that efficiently replicated and caused a cytopathic effect in Vero cells. Furthermore, after intracerebral inoculation, OZV replicates in BHK-21 and DH82 cells and causes high mortality in suckling mice (Ejiri et al., 2018). The first sighting of the *H. longicornis* tick that transmits BRBV was on a sheep in New Jersey, and later on, the tick was reported in New Jersey, as well as in the eastern United States and Arkansas (Rainey et al., 2018). Advanced diagnostic methods, such as the IgM antibody capture-enzyme-linked immunosorbent assay (MAC-ELISA) (Martin et al., 2000; Piantadosi and Kanjilal, 2020) and microsphere immunoassay (MIA) coated with recombinant viral antigens (Johnson et al., 2007; Basile et al., 2013), are recommended for BRBV detection. In severe cases, no specific treatments or vaccines are available to prevent BRBV infection; however, disease symptoms can be treated with an intravenous fluid infusion. Because the symptoms overlap (coinfections acquired *via* tick bites carrying BRBV), proper diagnosis is required to understand preventive strategies. Future research into the transmission and underlying pathogenic mechanisms of BRBV would aid in our understanding of their ecology.

### 2.1. Epidemiology

The BRBV was first isolated from a hospitalized 50-year-old man’s blood samples after he died in Kansas, United States Kosoy et al. (2015). Subsequently, two more deaths were reported from the BRBV (Miller, 2018; Vallbracht et al., 2018), and two humans recovered after treatment (Savage et al., 2017; Miller, 2018). The BRBV is genetically similar to the DHOV, isolated from a man in India (Bricker et al., 2019). A new *Oz-virus* isolated in Japan (Ejiri et al., 2018) revealed a close association with the previously identified BRBV (Fuchs et al., 2022). A study reported a high BRBV prevalence in raccoons (50%) and white-tailed deer (86%) in Missouri, USA (Jackson et al., 2019). During tick surveillance in Bourbon County and neighboring southern Linn County, 20,639 host-seeking ticks were collected for the detection of BRBV, and it was found that the detection of BRBV in humans after lone star tick *A. americanum* bites lends support to the theory that *A. americanum* serves as a vector for BRBV transmission to humans and wildlife. In a study, nucleotide sequences revealed similarities among *Thogotovirus* European, Asian, and African isolates (Kuno et al., 2001). A survey in Missouri, United States, discovered BRBV-neutralizing antibodies in human sera. Only three of the 440 human sera tested for BRBV were positive, indicating BRBV circulation in the region (Bamunuarachchi et al., 2022). In North Carolina, the detection of BRBV antibodies in white-tailed deer (Komar et al., 2020) aids in identifying new TBVs in the area (Hubálek and Rudolf, 2012).

### 2.2. Pathogenesis, and disease manifestations

According to research findings, *A. americanum* may be infected with the BRBV at the larva or nymph stages (Spare et al., 2021; Aziati et al., 2022). Multiple BRBV detections in *A. americanum* indicate that these viruses have similar life cycles and routes of transmission (Miller, 2018; Savage et al., 2018). *Thogotovirus* replication begins with the viral glycoprotein binding to the host cell’s receptors, followed by virus entry *via* clathrin-mediated endocytosis. BRBV is then transported to the endosome, where low pH triggers the viral genome toward the nucleus for transcription and replication (SIB, 2022). Understanding the pathogenesis of BRBV and the protective immune responses of the host cell is critical for disease prevention and control. BRBV can infect polarized human airway epithelia but not foreskin fibroblast cells because these cells may have a strong IFN response against the viral infection (Ryu et al., 2010), therefore, may be resistant to such virus infection. In addition, IFN-α2a and IFN-γ treatment elicits antiviral immunity that suppresses BRBV propagation (Fuchs et al., 2019). MRI findings show basal ganglia, brainstem, and cerebellum involvement in POWV-infected patients (Piantadosi et al., 2016). In contrast, DHOV-infected Institute of Cancer Research (ICR) mice provided overall pathological and immunological reactions to DHOV infection (Li et al., 2008). After DHOV infection, increased levels of pro-inflammatory cytokines and chemokines were found in the liver, lungs, and sera of mice (Li et al., 2008), providing a true alternative animal model for studying virus pathophysiology and potential treatment (Li et al., 2008). Adult mice infected with the DHOV intranasally, subcutaneously, or intraperitoneally developed fatal illnesses with obvious clinical and pathologic findings. In addition, hepatocellular necrosis and steatosis were found outside of the lungs, and widespread fibrinous necrosis in lymphoid organs was marked by severe lymphocyte loss, karyorrhexis, and neuronal degeneration in the brain. Because of its biosafety level 2 status and its pathological effect in mice, the DHOV may provide a low-cost, relatively safe, and realistic animal model for revealing viral pathogenesis and management studies (Mateo et al., 2007). *In vitro,* growth shows increased BRBV replication in a variety of cell lines, including monkey kidney cells (LLC-MK2 and Vero E6), frog (XLK-WG), hamster (BHK21Cl-15), duck embryo (DE), human (HeLa and HUH-7), Aedes, Anopheles (AP-61, C6/36, CCL-125, C7/10,), *Dermacentor* ANE58, DAE15, *Hyalomma*, and *Amblyomma* RAE/CTVM1, HAE/CTVM9 AVL/CTVM17 (Lambert et al., 2015). Infected individuals have experienced fatigue, nausea, fever, headache, vomiting, leukopenia, maculopapular rashes, and thrombocytopenia (Miller, 2018; Savage et al., 2018). Recently, the effective use of favipiravir medication in mice (Bricker et al., 2019), suggests a possible treatment for BRBV infection.

## 3. Powassan virus

POWV is a type of *tick-borne flavivirus* (TBFV), first isolated from a human who died from encephalitis in Powassan, Ontario, Canada, in 1958 (Goethert et al., 2021). Later, POWV was classified into two genetically and ecologically distinct lineages: Lineage-I (the prototype POWV) and Lineage-II (also known as the *deer tick virus*) (Goethert et al., 2021). The *flavivirus* family has an ssRNA genome (about 11 kb) with a 5′ UTR (loops A and B) and a 3′ UTR that becomes infectious when it enters the host cell (Choi, 2021). *Flavivirus* attaches its surface glycoprotein to the host cell receptors, such as heparan-sulfate, laminin-binding protein, etc. The fusion of virus-containing vesicles with endosomes causes nucleocapsid release into the cytoplasm, where viral genomic RNA is translated into a single polypeptide at endoplasmic reticulum-associated ribosomes. Cellular and viral proteases process the newly synthesized structural and non-structural proteins into new virions. The mature virions exit from the cell cytoplasm *via* exocytosis. Except for some morphological differences between tick cells and the membranous structures of infected vertebrate cells, virus replication in mammals and ticks exhibits similar mechanisms in the cytoplasm and the formation of membranous replication compartments (Mackenzie, 2005; Bollati et al., 2010). Except for a few human isolates, the majority of lineage-I isolates from northern America have come from *Ixodes cookei* and their hosts (such as groundhogs, skunks, or red squirrels; Mostafa and Dean, 2022), suggesting the need for a deep molecular-level investigation to understand their underlying association with ticks and vertebrate hosts.

### 3.1. Epidemiology

POWV Lineage-II has been primarily associated with *Ixodes scapularis* ticks and white-footed mice in northern and north-central America (Ebel et al., 1999; Brackney et al., 2008; El Khoury et al., 2013) before transmission to humans and other vertebrates. Evidence of POWV incidence has been reported in the United States (Goethert et al., 2021), Canada, Primorsky krai of Russia (L’vov et al., 1974; Leonova et al., 2009), Alaska and New Mexico (Deardorff et al., 2013). Between 2003 and 2018, over 106 POWV meningoencephalitis cases were reported, with a 15% mortality rate. The recent emergence of Powassan encephalitis in North America (Campbell and Krause, 2020) was most likely attributed to the POWV lineage-II transmitted to humans *via I. scapularis* bites (Goethert et al., 2021). A 50 to 59-year-old male from Windham, Connecticut, United States, was hospitalized with a known tick bite history and a central nervous system (CNS) illness. Although POWV, particularly lineage II, is rare, with only a few dozen cases reported in the United States each year, it is considered a serious public health threat due to its potential for severe illness and death (Ebel, 2010; Piantadosi et al., 2016; Mlera et al., 2017). POWV has also been linked to human disease in eastern Russia (Deardorff et al., 2013). Other closely related POWV serogroups were reported in Siberia, India, the United Kingdom, Ireland, Norway, Spain, Malaysia, and Saudi Arabia (Calisher et al., 1989). The post-infection complications involve damage to the patient’s CNS and other body organs (Goethert et al., 2021). Following the identification of the first human case, the geographic distribution of the POWV revealed high transmission with a higher fatality rate (over 30%) (Goethert et al., 2021). The adaptation of the POWV to invertebrate and vertebrate hosts directly influences the pathogen’s transmission in a specific area. Other factors, such as climate change, global warming, and agricultural practices, influence POWV’s geographic distribution (Lasala and Holbrook, 2010; Ostfeld and Brunner, 2015). Migratory birds may also aid in transmitting *Ixodes* species and their associated POWV over long distances (Waldenström et al., 2007).

### 3.2. Pathogenesis, and disease manifestations

POWV is transmitted through *Ixodes* species (*I. cookei*, *I. scapularis*, and *I. marxi*) after feeding on infected rodent blood (Ebel and Kramer, 2004). The primary POWV replication site is close to the tick bite, followed by the migration of the virus to lymph nodes *via* dendritic cells and macrophages, resulting in increased viral load/viremia in infected female BALB/c mice (Hermance et al., 2016). In the case of a neurotropic infection, POWVs can cross the blood–brain barrier and damage neurons (Belikov et al., 2014). A study investigated POWV pathogenesis using C57BL/6 mice. After footpad inoculation, infected mice exhibited severe infection with 100% mortality (Santos et al., 2016). In addition, immunofluorescence detected meningoencephalitis with mononuclear cell infiltration, activated microglia, and a polio-like disease. Moreover, the pathological analysis showed increased splenic macrophage counts, exploring the spleen’s role in POWV pathogenesis (Santos et al., 2016). A biopsy was performed to investigate the skin at the POWV-positive tick bite site. The findings showed that increased neutrophils, macrophages, dendritic cells, and mononuclear cells infiltrated the infected areas, resulting in a transient viremia stage that led to multiple organ dissemination (Hermance et al., 2016). The virus crosses the blood–brain barrier during the viremia stage. TNF-α may also be important in modulating the permeability of the POWV blood–brain barrier (Suthar et al., 2013). Although the exact underlying molecular mechanism of this blood–brain barrier crossing is unknown; however, brain capillary endothelium’s role in this mechanism cannot be ignored (Suthar et al., 2013). In the neurological stage of infection, POWV targets neurons and glial cells in the CNS (Kemenesi and Bányai, 2019). Surprisingly, rat astrocytes (a type of neuroglia in the CNS) resist POWV-mediated cell death by eliciting a pro-inflammatory IFN-α response, indicating dormant infection in rodent species (Potokar et al., 2019). The astrocyte’s resistance capacity may keep the enzootic transmission cycle going through long-term illnesses. POWV hemorrhagic disease with liver and spleen tropism can cause viscerotropic disease (Kemenesi and Bányai, 2019). *I. ricinus*-associated POWV modulates dendritic cell responses, pro-inflammatory cytokines, TNF-α, IL6, and immune cells at the biting site (Kemenesi and Bányai, 2019). The pattern-recognition receptors (PRRs) (host cells express helicases that function similarly to PRRs) and toll-like receptors recognize the POWV pathogen (Diamond et al., 2009; Clarke et al., 2021). These PRRs induce pro-inflammatory cytokines, IFN-α, and dendritic cells to mount an immune response against the invading POWV (Kawai and Akira, 2006; Kemenesi and Bányai, 2019). Viral recognition by dendritic cells regulates multiple pathways, including increased expression of MHC-II, T-lymphocytes (T helper and T cytotoxic cells), pro-inflammatory cytokines, and chemokines *via* molecular signaling (Masson et al., 2008; Diamond et al., 2009; Clarke et al., 2021), as determined in acute human infections (Blom et al., 2015). Another study reported a significant response of natural killer cells against POWV infection in humans (Blom et al., 2016). The antibody response in POWV diseases appears at the end of the viremia phase when IgM levels rise within 1–2 days of infection. On the other hand, IgM titers show a slight increase in secondary infections, whereas the IgG response is rapid and effective in eliminating POWV infection (Kemenesi and Bányai, 2019). Infected individuals experience flu-like symptoms, i.e., chills, headache, mild fever, sore throat, neurological complications, apnea, cognitive deficits, dysarthria, encephalitis, memory impairment, spasticity, and psychosis. Clinical diagnosis is generally based on symptoms, signs, and a person’s exposure history to ticks. According to serological diagnosis, a fourfold increase in POWV-specific IgG makes the presence of POWV in blood serum, tissue, or cerebrospinal fluid standard for laboratory diagnosis. IgM, which appears 6–14 days following infection, can be detected *via* ELISA (Kemenesi and Bányai, 2019). Further, the presence of POWV antibodies in patient serum at the CDC Laboratory in Ft. Collins, Colorado, emphasizes the need to adopt preventive measures to avoid infections (CDC, 2022). Due to the lack of effective medications/vaccines for Powassan virus prevention, people should be educated on how to avoid tick bites by treating their clothing with permethrin solution after outdoor activities in tick-endemic areas (Connally et al., 2019). People should also be educated on how domestic animals or pets can bring ticks into their homes, and a thorough tick inspection of animals after outdoor activities is recommended.

## 4. Omsk hemorrhagic fever virus

OHFV is a type of *flavivirus* that causes hemorrhagic fever primarily in the western Siberian region, including Omsk, Novosibirsk, Kurgan, and Tyumen (Růžek et al., 2010; Zhang et al., 2019). Ticks (*D. reticulatus*, *D. marginatus*, and *I. persulcatus*) transmit OHFV to humans and animals after feeding on infected hosts (Wagner et al., 2022). The *D. reticulatus* has evolved several extraordinary characteristics; for example, it has a high reproduction rate, a rapid life cycle completion, can survive underwater for months, and has a diverse host range (Földvári et al., 2016), which could enable OHFV transmission outside Russia (Wagner et al., 2022). In Russia, *Dermacentor* ticks serve as parasites that feed on small mammals, wild ungulates, domestic animals, and humans throughout their life cycle (Růžek et al., 2010), as shown in Figure 1C. According to evidence, ticks transmit OHFV to rodents (muskrats, narrow-skulled voles, and water voles) after feeding on an infected host. Moreover, OHFV has also been isolated from mosquitoes, *Coquilletidia richiardii*, *Ochlerotatus excruciates*, and yellow pygmy rice rat, *Oligoryzomys flavescens* (Wagner et al., 2022). Findings also show that OHFV isolated from different ticks and hosts differs from the early isolated strains (Růžek et al., 2010; Zhang et al., 2019).

### 4.1. Epidemiology

In mammalian cell lines, OHFV induces cytotoxic effects, i.e., changes in the cell through oxyphilic cytoplasmic inclusion formation (Růžek et al., 2010). It was speculated that the introduction of muskrats to Siberia might be responsible for the OHFV emergence, as the area was disease-free before (Růžek et al., 2010). Russians and Kazakhstanis have reportedly been aware of OFHV’s chronic and fatal infections (Ruzek et al., 2013; Wagner et al., 2022). Recently, Wagner and researchers reported OHFV incidence outside Russia (i.e., in the Republic of Kazakhstan) (Wagner et al., 2022). It was speculated that the virus could spread outside of endemic areas due to several factors, particularly climate change, shifting/migrating mammals, or birds that may carry tick vectors.

### 4.2. Pathogenesis, and disease manifestations

Although OHFV is potentially pathogenic in endemic areas, little is known about its underlying pathogenic mechanism or the immune response in infected individuals. Research findings show that the virus has a high affinity for hemopoietic (hematopoietic tropism) and vascular tissues (vascular tropism). However, *in vivo,* studies have investigated OHFV pathogenesis in laboratory mice and non-human primates. A study’s findings show that OHFV infection in BALB/c mice developed mild meningoencephalitis and significant cerebellar disorders (Holbrook et al., 2005), similar to symptoms previously described in OHFV-infected humans (Holbrook et al., 2005). The effects on the CNS and vascular systems were most prominent. Hemorrhages, focal glial cell proliferation, and perivascular inflammation infiltrate the brain parenchyma, causing CNS disorders (Holbrook et al., 2005). The OHFV infection did not cause paralysis or a fatal cerebrum infection. Distinct pathological findings in the OHFV-infected mice’s spleens revealed different immune responses, supporting the BALB/c mouse as an OHFV disease model (Holbrook et al., 2005). Generally, information is scarce on the impact of a persistent OHFV infection on its natural hosts. Laboratory tests detect a fatal neuro-infection (Shestopalova et al., 1972) in the susceptible wild muskrat (*Ondathra zibethicus*) after an extraneural or intracerebral injection (Kharitonova and Leonov, 1986). Other wild vertebrates show mostly asymptomatic diseases with viremia (Kharitonova and Leonov, 1986; Růžek et al., 2010). Some infected animals showed poor mobility, weakness, and hyperpnoea. In addition, the cerebellum and brain hemispheres accumulated the highest viral load, followed by the lungs, kidneys, blood, feces, spleen, and liver (Shestopalova et al., 1972). The viral persistence in infected subcutaneous tissues and internal organs is a defining characteristic of the pathogenesis processes in mice. In severe infections, OHFV may cross the blood-barrier and infiltrate the brain, resulting in increased INF production in the CNS of baby mice (Hofmann et al., 1969). Further, edema in the infected mice causes sensory disturbances that may lead to collapse and shock (WHO, 1985), which may also increase the host’s susceptibility to other pathogens (Novitskiy, 1948). In a study, OHFV-infected mice developed pneumonia, paralysis (Chumakov, 1948), meningoencephalitis with a high viral load in the cerebellum, and other pathological findings such as enlarged spleens and impaired gastrointestinal tracts, kidneys, and liver (Růžek et al., 2010; Li et al., 2021). In contrast, the infected macaques (*Macaca radiata*) showed no obvious pathological or histological symptoms, and no virus was isolated from their blood. Furthermore, high aminotransferase levels were observed, indicating viral replication in the liver (Kenyon et al., 1992). Direct contact with an infected animal’s blood, body fluids, virus-carrying tick bites, or the feces/urine of an infected or dead rodent can result in chronic infection (Ruzek et al., 2013; Wagner et al., 2022). There has been no evidence of person-to-person transmission so far. High-grade fever, headache, cough, myalgia, moderate to severe hemorrhagic manifestations, pneumonia, nephrosis, and meningitis are all symptoms of OHF, with a mortality rate ranging from 0.4 to 3% (Kharitonova and Leonov, 1986; Růžek et al., 2010; Zhang et al., 2019). Currently, no specific treatment or vaccine is available to treat or prevent OHF; however, symptoms can be treated to minimize disease manifestations (Orlinger et al., 2011). Based on the antigenic similarity, available tick-borne encephalitis (TBE) vaccines were used as a preventive measure against OHFV during the 1991 outbreak. However, no clinical data support the vaccine’s efficacy for complete prevention (Růžek et al., 2010). General disease management strategies include early hospitalization, drinking more water, and a nutritious diet supplemented with potassium, chloride, and vitamins K and C, which are recommended for quick recovery. OHF can be diagnosed based on clinical and epidemiological observations. ELISA is crucial for the accurate diagnosis of antibody titers in OHFV patients’ serum samples following 1–3 weeks of infection (Růžek et al., 2010; Orlinger et al., 2011).

## 5. Kyasanur forest disease virus

The KFDV is a TBFV that spreads from infected mammals, notably monkeys, to humans *via H. spinigera* bites. The KFDV transmission cycle involves multiple *Haemaphysalis* species, notably *H. spinigera*, some *Ixodes* species, and hosts, such as bats, birds, monkeys, rodents, and shrews (Pattnaik, 2006). KFD disproportionately impacts low-income rural communities in India, including forestry workers and smallholder farmers who harvest non-timber forest products (Pattnaik, 2006; Burthe et al., 2021), which may be attributed to their intervention in the natural ecosystem. However, no study confirms direct virus transmission from human to human (Shah et al., 2018; Bhatia et al., 2020). According to experimental evidence, adult female ticks transmit the KFDV to their offspring (Matser et al., 2009; Burthe et al., 2021).

### 5.1. Epidemiology

In southern India, this TBV causes a devastating, deadly hemorrhagic disease with approximately 500 cases and a 10% mortality rate per year (Pattnaik, 2006). KFDV was first isolated from a sick monkey in the Kyasanur Forest in Karnataka State, India, in 1957. KFDV is carried by ticks, Hemaphysalis spinigera, after being bitten by an infected tick, rodents, shrews, and monkeys, causing epizootics with high fatality in primates (Pattnaik, 2006; Burthe et al., 2021). Asymptomatic infections result in KFDV-specific antibody responses (Stone, 2014). Ticks also transmit the virus between the co-feeding ticks, i.e., without involving hosts (Randolph, 2011). Several reports have discussed the route of KFDV transmission from tick to human after being bitten or having direct contact with an infected *Haemaphysalis* species (becomes infected by feeding on viral reservoirs). Humans are merely incidental hosts for the KFDV and play no role in disease transmission (Matser et al., 2009), representing the KFDV as a spillover pathogen from wildlife reservoirs. However, no empirical data exist that describe the role of tick species that transmit KFDV to their hosts (Burthe et al., 2021). This virus affected those who visited the forests to cut wood, graze cattle, and engage in other agricultural-related activities. In the early stages of the disease, insect collectors on their way to investigating outbreaks become infected. Since 2005, KFD cases have increased, with recent outbreaks in neighboring Karnataka districts like Chikamagalur, Dakshina Kannada, Uttar Kannada, and Udupi, Karnataka, India (Gurav et al., 2018). The disease has now spread to Maharashtra, Kerala, Tamil Nadu, and Goa because of deforestation, agricultural use of forestlands, and subsequent displacement of land and animals (Burthe et al., 2021). A household survey of smallholder farmers, forest users, and native tribes in KFD-affected regions revealed that 69% of the respondents were concerned about the disease’s repercussions, highlighting it as a major public health issue (Asaaga et al., 2021).

### 5.2. Pathogenesis, and disease manifestations

Humans, wild rodents (i.e., shrews, squirrels, white-bellied/white-tailed rats), bats, black-faced langurs, bonnet macaques, grey langurs, ground-nesting birds, Indian crested porcupines, cattle, and gerbils are all susceptible to the KFDV infection (Sreenivasan et al., 1986; Muraleedharan, 2016; Bhatia et al., 2020). Infected macaques exhibit leucopenia, thrombocytopenia, and high levels of alkaline phosphatase, alanine transaminase, and nuclear material phagocytosis in the peripheral-endothelial system. KFDV causes hemorrhagic disease, implying the presence of molecular determinants within the genome linked to viral pathogenicity. The E protein is a critical causal element of tissue tropism, mediating virus entry into the host cell and is responsible for virus immunogenic and phenotypic properties, thereby playing important roles in pathogenesis and immune evasion (Barker et al., 2009). The virus targets the endothelial cells lining the blood vessels, causing endothelial damage and leakage, bleeding, organ damage, and even shock in severe cases. High fever (lasting 6–11 days), anorexia, abdominal pain, cough, body ache, diarrhea, headache, insomnia, leucopenia, myalgia, thrombocytopenia, increased intravascular permeability, prolonged clotting time, bleeding from the gums/nose (Shah et al., 2018; Bhatia et al., 2020; Gupta et al., 2020), decreased heart rate, elevated creatinine phosphokinase, and elevated blood urea nitrogen levels are among the KFD symptoms (Bhatia et al., 2020). The infected person may also express reduced eosinophils, neutrophils, and lymphocyte counts within the first week of illness. About 10–20% of KFDV patients experience a secondary phase relapse (Muraleedharan, 2016), characterized by neurological disorders, including confusion, convulsions, drowsiness, tremors, etc. KFDV infection is diagnosed based on clinical symptoms and confirmed by laboratory tests. Virus isolation is the gold standard for diagnosing KFDV infection but it is risky and expensive. Molecular diagnosis, particularly the reverse transcription loop-mediated isothermal amplification (RT-LAMP) assay, is a quick, sensitive method for detecting KFDV infection and would be useful for high throughput screening of clinical samples (Kumar et al., 2021). The Indian Council of Medical Research developed the first vaccine to prevent KFDV (Bhatia et al., 2020); although the vaccine is safe, but has a low efficacy rate in humans (only 62%) (Kasabi et al., 2013). KFDV case is not treatable with antiviral medications; however, disease complications can be treated based on the signs and symptoms. Supportive therapies, such as intravenous electrolyte transfusions and maintaining oxygen levels and blood pressure, are suggested to manage patient health status (Bhatia et al., 2020).

## 6. Colorado tick fever virus

The CTFV member of the *Reovirales* order, *Spinareoviridae* family, and *Coltivirus* genus comprises a 12-segmented dsRNA genome (Owen et al., 2021). The CTFV genome is approximately 29,000 base pairs in size and contains an icosahedral capsid of about 80 nm in size (CDC, 2021a). CTF is a rare viral disease spread in humans by CTFV-infected Rocky Mountain wood tick (*D. andersoni*) bites (CDC, 2021a). Ticks become infected after feeding on the blood of host animals, such as rodents, squirrels, chipmunks, etc. Other animals, such as elk, marmots, and deer, can also be infected by *D. andersoni* (CDC, 2021a).

### 6.1. Epidemiology

Infected *D. albopictus*, *D. arumapertus*, *D. andersoni*, *D. occidentalis*, *Haemaphysalis leporispalustris*, *Otobius lagophilus*, *I. sculptus*, and *I. spinipalpis* may also transmit the CTFV to animals (Attoui et al., 2005); however, there is no evidence of CTFV transmission from person to person. Virus transmission *via* blood transfusion cannot be ignored due to the evidence that CTFV can live for several months in human and animal red blood cells, suggesting that blood donation should be avoided for at least 6 months after CTFV infection (CDC, 2021a). Approximately 400 CTF cases have been reported each year, with 0.02 cases per 1 million people between 2002 and 2012 (Yendell et al., 2015), whereas only 59 cases were reported to the CDC from 2010 to 2019 (CDC, 2021a). As of September 2020, CTF cases were reported in Arizona, Colorado, Dakota, Idaho, New Mexico, Montana, Oregon, South Utah, and Wyoming (CDC, 2021a). In addition, CTFV has been reported in Canada (British Columbia and Alberta).

### 6.2. Pathogenesis, and disease manifestations

The incubation period, i.e., the time between a tick bite and the onset of illness, can range from 1 to 14 days (Romero and Simonsen, 2008; Burrell et al., 2016). Hospitalization is required in approximately 20% of severe CTF cases with hemorrhagic fever, meningitis, and meningoencephalitis (Romero and Simonsen, 2008; Burrell et al., 2016). In children, fatal CNS or hemorrhagic complications are commonly attributed to intravascular coagulopathy (Bennett et al., 2019), bleeding, and shock (Romero and Simonsen, 2008), with 5 to 10% of deaths. Persistent viremia is caused by virus replication in erythroblasts, reticulocytes, and CD34+ hematopoietic cells (Attoui et al., 2005). According to a survey, only 6.6% of *D. andersoni* in Western Montana were infected with CTFV (Williamson et al., 2019), indicating the virus’s persistent infection by targeting skin endothelial cells (Owen et al., 2021). Endothelial cells play an essential role in homeostasis, regulation, and molecular trafficking by controlling vascular permeability and coagulation (Mai et al., 2013). In addition, endothelial cells facilitate immune cell trafficking, antigen presentation, and the production of increased pro-inflammatory cytokines and chemokines. In the case of severe infections, the induced pro-inflammatory cytokine/chemokine levels, innate immune response, and inflammatory response may cause endothelial cell injury, vascular dysfunction, or even patient death (Owen et al., 2021). *In vitro*, CTFV caused cytopathic effects on BHK21 cell lines, KB cell lines, glial progenitor cell lines, and hematopoietic stem cell lines (Owen et al., 2021). A study found that after CTFV infection, HMEC-1 human cell lines died *via* an apoptotic-like pathway that was not dependent on caspase-3/7 activity. Furthermore, in the presence of caspase inhibition, the HMEC-1 accumulated fewer viruses (Owen et al., 2021). CTF symptoms are typically flu-like, with fever, headache, muscle aches, and fatigue, but the illness can progress to more serious complications such as meningitis or encephalitis (Romero and Simonsen, 2008; Burrell et al., 2016). Serological tests are typically used in laboratory diagnosis, and supportive treatment is required. Although CTFV infection cannot be treated due to a lack of specific viral medications or vaccines, the risk of infection can be reduced by taking preventive measures (Burrell et al., 2016; CDC, 2021a), such as avoiding unnecessary exposure to tick-infested areas (WDH, 2022).

## 7. Crimean-Congo hemorrhagic fever virus

CCHFV, which causes CCHF, is one of the most severe viral diseases in humans. The virus is transmitted *via* ixodid ticks, particularly those of the genus *Hyalomma*, and is initially characterized by unspecific signs and symptoms (Rathore et al., 2021). The CCHFV genome is a tri-segmented, negative-sense ssRNA with a conserved 3′ and complementary 5′ ends, allowing the RNA to form a looped structure. The small (S) segment (∼1.6 kb in size) encodes the multifunctional nucleocapsid (N) protein, which is an important virion structural component and is involved in viral infection. The medium (M) segment (∼5.4 kb in size) encodes a glycoprotein precursor, which is further processed into glycoprotein-N (Gn), glycoprotein-C (Gc), and other non-structural proteins. CCHFV envelope glycoproteins have a crucial role in virus formation, RNA binding, oligomerization, and infectivity (Erickson et al., 2007). CCHFV invades the host cell through clathrin-mediated endocytosis and fuses with endosomal membranes (Bertolotti-Ciarlet et al., 2005). A recent study found that a single amino acid substitution in the CCHFV glycoprotein significantly reduced viral fusion with human cells, indicating that a glycoprotein variant in ticks may reduce viral infectivity in humans (Hua et al., 2020). This suggests that extensive CCHFV genome sequence variability may contribute to divergent clinical outcomes. In addition, to tick bites, CCHFV can be transmitted to humans through direct contact with infected animal tissues or other bodily fluids (i.e., during slaughter, handling of meat, or caring for infected patients). Furthermore, nosocomial outbreaks in healthcare settings have been reported in which infected blood or bodily fluids were handled incorrectly, resulting in human-to-human transmission (Tsergouli et al., 2020).

### 7.1. Epidemiology

CCHF transmitted *via Hyalomma* species, is widely distributed in Asia (Iraq, Iran, India, China, Pakistan, Afghanistan, Russia, Tajikistan, Uzbekistan, Kazakhstan, Kuwait, Oman, UAE, and Saudi Arabia) (Al-Abri et al., 2017), Africa (Sudan, Kenya, Egypt, Nigeria, Senegal, Chad, Uganda, Tanzania, South Africa, Mauritania, and Congo) (Temur et al., 2021), and Europe (Albania, Bulgaria, Turkey, Greece, Georgia, and Spain) (Maltezou et al., 2010; Figure 4B). In humans, the time required for the virus infection to cause hemorrhage is approximately 5–6 days. The first case of CCHFV was reported in a 51-year-old man in Tintane, Assaba, Mauritania, who had abdominal pain, diarrhea, vomiting, a 41°C body temperature, epistaxis, gingivorrhagia, diffuse ecchymosis, severe anemia, thrombocytopenia, etc. (Boushab et al., 2020). Later, an unusual CCHF case was reported in Nouakchott, Mauritania (Kleib et al., 2016), raising the prospect of the virus spreading from Mauritania to West Africa (Kleib et al., 2016). These findings, combined with CCHF cases and other findings in neighboring countries such as northwestern Senegal (Tall et al., 2009) and Mauritania (Kleib et al., 2016), suggest that CCHF is endemic in West Africa, emphasizing accurate disease diagnosis and raising awareness. CCHF was first described in Turkey in 2002, and more than 9,000 human cases were confirmed in 2015. During the COVID-19 pandemic, another CCHF outbreak occurred in Turkey (Leblebicioglu et al., 2016). Later, the Turkish Ministry of Health confirmed 243 CCHF human cases with 13 deaths (Infectiousdiseasenews, 2021). Most of the CCHF cases were reported in northern, eastern, and central Anatolia, where agricultural hubs exist, as well as the central Black Sea region (Daily-Sabah, 2022). Apart from disease awareness campaigns in areas where cases are more common, the State Ministry of Agriculture and Forestry initiates programs to eradicate the tick *Hyalomma marginatum* in endemic regions. From January 1 to May 22, 2022, Iraq’s health authorities notified the WHO of 212 cases of CCHF, of which 115 were suspected, and 97 polymerase chain reaction (PCR) confirmed cases, with an overall of 27 deaths (14 suspected and 13 PCR-confirmed cases). These reported cases are significantly higher than the 33 PCR-confirmed reported cases in 2021. Cases have been reported in several areas of Iraq, and the outbreak may put additional strain on an already overburdened healthcare system. Most confirmed cases had a history of direct contact with animals, including butchers and livestock breeders. Over half of the confirmed cases were among males aged 15–44 years (WHO, 2022c). Travelers from CCHF endemic countries were responsible for transmitting the virus to the non-endemic regions of the United Kingdom (for example, imported from Bulgaria and Afghanistan) (Chamberlain et al., 2013; Lumley et al., 2014), France (tourists from Senegal) (Tall et al., 2009), Germany (an American soldier returning from Afghanistan) (Conger et al., 2015), and a traveler from Bulgaria (Olschläger et al., 2011). In Iran, CCHF cases have been reported in almost every province. In a study, four sheep (three from Gachsaran and one from Dena regions) reported positive for CCHFV IgG, indicating virus circulation in Kohgiluyeh and Boyer-Ahmad Province, Iran. This finding suggests comprehensive monitoring programs to understand the CCHFV’s circulation in ticks and host animals (Ghasemian et al., 2021). In 1998, 19 confirmed CCHF cases were reported in northeastern Afghanistan, making them the country’s first documented cases. Later, an outbreak was reported in Afghanistan in 2008 (Mofleh and Ahmad, 2012). Regarding seasonal prevalence, the highest CCHF cases found from June to September probably coincide with Eid-al-Adha, with 13.7% of butchers and 11.8% of shepherds being two high-risk occupations in Afghanistan for CCHFV infection (Qaderi et al., 2021). ELISA and immunofluorescence assays detected CCHFV-specific IgG in 19% of sheep and 5% of goat serum samples in Balochistan, Pakistan (Kasi et al., 2020). In addition, this cross-sectional study also identified potential risk factors for CCHF seropositive sheep and goats. For example, open housing systems, grazing, vegetation in or around the yard, a lack of tick preventive measures, rural poultry absence, and tick-infested animals were all significant risk factors for CCHFV. Identifying risk factors may aid in lowering the CCHFV infection risk in cattle and humans (Kasi et al., 2020). A recent study examined the seroprevalence of CCHFV in sheep sera (*n* = 176) and found that CCHFV-specific antibodies were detected in 17 sera, indicating CCHFV circulation in the westernmost region of the Balkans, Bosnia and Herzegovina, which raises concerns about CCHFV transmission out of this region (Satrovic et al., 2022). A cross-sectional survey tested 800 humans, 666 cattle, 549 goats, and 32 dogs’ sera samples from the Ugandan cattle corridor for CCHFV-specific IgG antibodies. Results show 221/800 CCHFV seropositivity in humans, 612/666 in cattle, 413/549 in goats and 18/32 in dogs. Human seropositivity was significantly associated with livestock farming, age, and collecting/eating engorged ticks. High CCHF seropositivity among livestock farmers, including collecting/eating engorged ticks, highlights the need for further surveillance and disease control strategies (Atim et al., 2022).

### 7.2. Pathogenesis, and disease manifestations

The first contact between the tick-host occurs during the tick’s bite or feeding on the host. The tick secretes saliva into the feeding lesion, *via* which pathogens mediate vertebrate host inflammatory and immune responses (Kaufman, 1989). Despite the host’s inflammatory and immune responses, the tick attaches to hosts for blood-feeding *via* the pharmacy located in its salivary glands (Connolly-Andersen et al., 2007). CCHFV Gn and Gc help their attachment to the target cell surface receptors. An interaction between viral Gn/Gc and target cell surface protein (i.e., nucleolin found within nucleoli may work as a putative entry factor) (Xiao et al., 2011) demands deep investigations to unveil the role of nucleolin in CCHFV internalization. CCHFV enters the cells using clathrin and the clathrin pit adaptor protein-2 complex (Simon et al., 2009; Garrison et al., 2013). CCHFV particles enter the cell and travel to endosomes and vesicular bodies, where the virus envelope fuses with cellular membranes. Cellular microtubulin and actin filaments are essential for CCHFV internalization, replication, and progeny production (Andersson et al., 2004; Simon et al., 2009). It has also been shown that the virus Gn interacts with cellular chaperones of the heat shock protein 70 families in association with DnaJ cofactor adapter proteins, which play roles in folding and transporting newly synthesized proteins (Surtees et al., 2016). Aquaporin 6 protein (a channel that facilitates water and small solutes across the plasma membrane) has also been shown to be involved in CCHFV replication (Molinas et al., 2016). DC-SIGN (C-type lectin) expressed on the surface of antigen-presenting dendritic cells probably acts as an entry factor for CCHFV (Suda et al., 2016). CCHFV highly replicates in epithelial cells, dendritic cells, and tissue-resident macrophages at the inoculation site. Apart from increased macrophage counts, more natural killer cells, CD4+, and CD8+ lymphocytes have been observed in CCHFV infections (Yilmaz et al., 2008; Akinci et al., 2009). As the disease progresses, the uncontrolled apoptosis of T lymphocytes contributes to lymphopenia (reduced lymphocyte count) in a STAT-1 knockout mouse model (Bente et al., 2010). These cells facilitate virus spread and infect local lymph nodes and peripheral blood-borne monocytes (Connolly-Andersen et al., 2007; Akıncı et al., 2013). The infection of endothelial cells and peripheral blood-borne monocytes results in extravasation into parenchymal tissue, enabling virus interaction with basolateral cell receptors (Connolly-Andersen et al., 2007). Secondary replication in these body parts facilitates systemic virus spread (Akıncı et al., 2013), as demonstrated by an experimental animal model: viral replication occurs in the blood on the first day of infection, then in the spleen and liver on the second day, and then spreads systemically to the lungs, kidneys, and brain (Bente et al., 2010). Endothelial cell damage is responsible for cellular hemostasis failure by platelet stimulation and degranulation as well as activation of the intrinsic coagulation cascade in host organs (Weber and Mirazimi, 2008; Bodur et al., 2010). It has been shown that CCHFV non-structural proteins may induce apoptosis *via* both intrinsic and extrinsic pathways (Barnwal et al., 2016). The induced apoptosis was attributed to releasing cytokines and chemokines from CCHFV-infected cells (Weber and Mirazimi, 2008; Barnwal et al., 2016). *In vitro* study showed that RIG-I acts as a PRR for CCHFV and mediates type I IFN response (Spengler et al., 2015). Tick cell lines enable CCHFV studies to offer an alternative approach to understanding tick cells’ response to invading viruses (Bell-Sakyi et al., 2012). *In vivo*, CCHFV transmission has been established using IFN knockout mice, an additional tool for studying tick-pathogen interactions(Gargili et al., 2013). CCHF manifestations include high fever, myalgia, dizziness, neck pain and stiffness, backache, headache, sore eyes, nausea, vomiting, diarrhea, abdominal pain, thrombocytopenia, leukopenia, and elevated liver enzyme levels. After a few days, the agitation may be replaced by sleepiness, depression, and lassitude, and the abdominal pain may localize to the upper right quadrant, with detectable liver enlargement. Patients may also experience a fast heart rate, enlarged lymph nodes, and a petechial rash on internal mucosal surfaces. There is usually evidence of hepatitis; severely ill patients may experience rapid kidney deterioration and sudden liver or pulmonary failure following an illness. The CCHF mortality rate is approximately 30%, with deaths occurring in the second week of illness (Yakut et al., 2021; WHO, 2022a). In brief, over the last decade, considerable progress has been made in exploring cellular components involved in tick-host-pathogen interactions; however, there is limited knowledge in the case of CCHFV due to its high-level containment requirement. Identifying the underlying molecular drivers that promote CCHFV survival in tick vectors may allow us to disrupt these processes and reduce tick populations and CCHF prevalence (de la Fuente et al., 2017). Moreover, the identification of the signaling pathways taking place during CCHFV-host interactions provides the opportunity to design novel control strategies for CCHF.

## 8. Heartland virus

The genus *Bandavirus*, classified under the order *Bunyavirales*, family *Phenuiviridae*, including HRTV, *Dabie bandavirus* (SFTSV), etc., poses threats to public health. The genus *Bandavirus* contains viruses that comprise three segments, i.e., small (S ~ 1.7 kb in size), medium (M ~ 3.2 kb in size), and large (L ~ 6.4 kb in size) (Adams et al., 2017). The M segment encodes Gn and Gc, which are crucial for the viral interaction with host receptors to neutralize host immune responses (Walter and Barr, 2011). The S segment encodes the structural protein nucleocapsid (N). In addition, the L segments synthesize NS proteins (RNA-dependent RNA polymerase). The viral Gn and Gc fused with the host cell’s DC-SIGN, heparan-sulfate, and non-muscle myosin heavy-chain IIA receptors. This interaction is mediated by the viral Gc and is a critical step for pathogenesis. HRTV replication is complete in the host cell’s cytoplasm and begins with the release of ribonucleoproteins, followed by the synthesis of new viral RNA. Ribonucleocapsids are formed in the Golgi apparatus, and progeny virions are exocytosed *via* the plasma membrane (Spiegel et al., 2016).

### 8.1. Epidemiology

HRTV transmitted by tick bites (*A. americanum*) was first reported in northwestern Missouri in 2009 (Pastula et al., 2014). In 2010, a tick-borne *phlebovirus* caused similar symptoms in China, Korea, and Japan, whereas a bat-borne, *Malsoor virus*, was responsible for HRTV-like symptoms in western India (Mourya et al., 2014). In 2018, two HRTV-infected cases were reported in Illinois, United States (McMullan et al., 2012). HRTV is closely related to the *Albatross Hunter Island virus*, isolated from *Ixodes* species and dying sea birds in Australia (Gauci et al., 2015). As of January 2021, more than 50 HRTV cases had been reported in midwestern regions (such as Illinois, Indiana, and Iowa), southwestern (Arkansas), southeastern (Georgia, Kentucky, North Carolina, and Tennessee), midwestern (Kansas and Missouri), and south-central (Oklahoma) of the United States. It has been shown that all local populations and visitors to HRTV endemic areas are at increased risk (Tuten et al., 2020; CDC, 2021b). Most patients reported seeing ticks (*A. americanum*) on their bodies 2 weeks before becoming ill (Brault et al., 2018). A study reported *A. americanum’s* presence on the bodies of two HRTV-positive adult males (Tuten et al., 2020), indicating that the virus is widespread in these ticks in Illinois. The emergence of HRTV is a significant concern because of the geographic distribution and increased abundance of *A. americanum* (Tuten et al., 2020). Some HRTV patients have a high viral load in their bloodstream, indicating that healthy people in contact with patient blood are at an increased risk of getting infections (Brault et al., 2018). HRTV case mortality has ranged from 13 to 16%, with the highest rate occurring in 50-year-old men with sepsis and multiple organ failure (Fill et al., 2017). The first fatal case was an 80-year-old Tennessee man who died after being hospitalized with fever, leukopenia, thrombocytopenia, organ failure, and bleeding. In the post-mortem, HRTV was detected in the spleen and lymph node samples (Muehlenbachs et al., 2014).

### 8.2. Pathogenesis, and disease manifestations

HRTV disease symptoms are very similar to tick-borne ehrlichiosis, transmitted *via* the same tick (*A. americanum*). For example, two HRTV-infected farmers in northwestern Missouri developed ehrlichiosis-like mild fever, leukopenia, and thrombocytopenia (Pastula et al., 2014). A study injected HRTV into animals to assess potential vertebrate hosts and establish a tick-vertebrate model for evaluating vector competence. Seronegative raccoons from various fields in the Midwestern United States were challenged with HRTV and produced a high rate of neutralizing antibodies (Bosco-Lauth et al., 2015; Riemersma and Komar, 2015). None of the susceptible raccoons demonstrated disease signs or a detectable HRTV load (Bosco-Lauth et al., 2016). In a similar experiment, naive goats exposed to SFTSV experienced high viral loads (Jiao et al., 2015). In contrast, goats exposed to SFTSV-infected ticks and virus-infected needles failed to develop detectable viremia (Jin et al., 2012). In an experiment, goats were exposed to SFTSV-infected ticks, suggesting that a long-term vertebrate host’s exposure to ticks could result in high viral loads sufficient to infect other ticks (Jin et al., 2012). A study also found that after being inoculated with HRTV, experimental mouse models failed to develop lesions or detectable viremia. In addition, HRTV-infected chickens, rabbits, and hamsters could not produce antibodies against the HRTV infection (Bosco-Lauth et al., 2016). This study further reports that when outbred CD-1 mouse models (immune-competent mice) were inoculated with HRTV, they failed to demonstrate neurovirulence (Bosco-Lauth et al., 2016). A dose-dependent response was observed in INF-type-I and -type-II deficient mice when a lethal dose of 50% of 10 plaque-forming units of HRTV was administered intraperitoneally, given that the low feeding efficiency of ticks in immune-compromised mice may differ from that of HRTV-infected vertebrates (Bosco-Lauth et al., 2016). On the other hand, SFTSV-inoculated immune-competent mice exhibit pathological abnormalities similar to those seen in HRTV patients with leukopenia, thrombocytopenia, and elevated liver transaminases. These manifestations were mild, and the SFTSV replication was restricted to the spleen (Jin et al., 2012), indicating a protective INF response within mouse models. Hamsters deficient in INF type-I signaling were susceptible to HRTV lethality, indicating that rodents’ protective INF response against HRTV infection was well-established (Westover et al., 2017). *Phleboviruses* infect mononuclear cells, eliciting strong INF-type-I reactions (Wuerth and Weber, 2016). More research is needed to determine these cells’ direct and indirect roles in HRTV trafficking within hosts and their precise roles in pathological abnormalities in humans. Fever, fatigue, anorexia, diarrhea, leukopenia, thrombocytopenia, and elevated liver transaminases were all symptoms of HRTV infection (McMullan et al., 2012). Other disease symptoms are headaches, nausea, myalgia, arthralgia, and weakness. Some patients reported a local rash frequently associated with the initial tick bite (Brault et al., 2018). Patients with fever, leukopenia, and thrombocytopenia should seek laboratory testing in *A. americanum* endemic areas (Staples et al., 2020).

In brief, the evidence presented above shows that ticks transmit the most diverse array of infectious agents to animals and humans. Thus, there is a need to explore the global distribution of ticks that transmit infectious diseases to humans and animals. Further, there is a need to develop the latest novel diagnostic tools and treatment options to control these diseases. Interestingly, clinical manifestations in infected individuals highlight distinct features of each virus, such as thrombocytopenia in BRBV, CCHFV, HRTV, and KFDV. In some cases, TBV RNA and their associated induced IgM and IgG antibodies can be detected in infected individuals with leukopenia, thrombocytopenia, and mild to moderate elevations of liver transaminases (CDC, 2019; Madison-Antenucci et al., 2020). There is a need to develop novel *in vitro* and *in vivo* models to understand TBV’s underlying pathogenic mechanisms, which will ultimately help with disease prevention and control measures.

## 9. Conclusions and future perspectives

Ticks transmit increased numbers of viral pathogens, such as BRBV, POWV, OHFV, CTFV, CCHFV, HRTV, and KFDV, which constantly threaten human and animal life worldwide, particularly those doing outdoor activities, increasing contact with ticks, their potential hosts. Ticks carrying viruses or their infected hosts’ migration from endemic areas may increase the risk of infections in humans and animals. Therefore, identifying ticks and their associated pathogens will be of great importance in preventing TBV diseases.

Physicians should consider TBV infections in differential diagnostics in endemic regions, especially locally active TBVs, as most viruses are geographically restricted. Diseases occurring in remote areas should be reported to the local health department, which can issue preventive measures against ticks and their associated viruses. People should avoid contact with sick animals, particularly bats, muskrats, and monkeys, and avoid drinking non-pasteurized goat milk. The public should also be encouraged to report dead or sick animals, particularly muskrats or monkeys, as an early warning for TBV infections (WHO, 1985). In addition, short-term efforts should increase public awareness of the BRBV, POWV, CTFV, CCHFV, HRTV, and KFDV-associated illnesses. People should avoid unnecessary visits to tick-endemic areas. In case of visiting, certain preventive measures should be taken, such as wearing long-sleeved nylon clothes (WHO, 1985), apart from applying tick repellants containing n-n-diethylmetatoluamide and permethrin to the skin, clothes, and other items (Pages et al., 2014). Wearing light-colored clothing is suggested as an option for observing crawling ticks on the body before returning from tick-endemic areas. To prevent ticks from crawling up inside, pants legs should be tucked into socks, and long-sleeved shirts should be tucked into pants. Furthermore, spraying permethrin-containing repellents on boots and clothing can deter tick attachment (WDH, 2022). It has strongly encouraged people to seek medical attention when they notice a tick on their body parts or experience their first sign in endemic areas.

Improved healthcare infrastructure, new diagnostic tools, and trained personnel should be deployed in tick-endemic areas for disease control. Seasonal migratory birds, animals, or mammals may transmit ticks or TBVs across many regions and countries, suggesting that advanced research will aid in developing surveillance systems (such as radio-tracking) to track the emergence of TBVs. Surveillance programs should also identify relevant animals or mammals that help the tick vectors complete their life cycle. For example, birds and bats have gained attention due to their role in spreading ticks and their associated viruses during seasonal migration (Calisher et al., 2006; Buczek et al., 2020). Humans traveling with ticks or viruses from endemic areas have also gained attention for spreading TBV diseases (Tall et al., 2009; Lumley et al., 2014; Conger et al., 2015). Therefore, long-term efforts should be directed toward various parameters influencing the ecological distribution of ticks to investigate global disease circumstances and countermeasures. There is an urgent need to develop new vaccines/drugs and make them available to inaccessible remote tick endemic areas for public immunization and disease management.

## Author contributions

TS and XX conceptualized the study. QL conducted the database search. TS, XX, BW, and ZB narratively screened the databases, then extracted their data and prepared the initial draft of the manuscript. TS prepared the final draft of the manuscript. XX supervised the project and critically assessed the manuscript. All authors contributed to the article and approved the submitted version.

## Funding

This study was supported by the Yunnan Key R&D Program (No. 202103AQ100001) and Yunnan Major Scientific and Technological Project (No. 202202AG050013).

## Conflict of interest

The authors declare that the research was conducted in the absence of any commercial or financial relationships that could be construed as a potential conflict of interest.

## Publisher’s note

All claims expressed in this article are solely those of the authors and do not necessarily represent those of their affiliated organizations, or those of the publisher, the editors and the reviewers. Any product that may be evaluated in this article, or claim that may be made by its manufacturer, is not guaranteed or endorsed by the publisher.
